# Levels of Cyclooxygenase 2, Interleukin-6, and Tumour Necrosis Factor-*α* in Fibroblast Cell Culture Models after Photobiomodulation at 660 nm

**DOI:** 10.1155/2021/6667812

**Published:** 2021-02-13

**Authors:** Asma Shaikh-Kader, Nicolette N. Houreld, Naresh K. Rajendran, Heidi Abrahamse

**Affiliations:** Laser Research Centre, Faculty of Health Sciences, University of Johannesburg, Johannesburg 2028, South Africa

## Abstract

Chemicals and signaling molecules released by injured cells at the beginning of wound healing prompt inflammation. In diabetes, prolonged inflammation is one of the probable causes for delayed wound healing. Increased levels of cyclooxygenase-2 (cox-2), interleukin–6 (IL-6), and tumour necrosis factor-alpha (TNF-*α*) are associated with the inflammatory response and in diabetes, and increased levels of these contribute to chronic wounds that do not heal. Rising levels of cox-2, IL-6, and TNF-*α* have also been associated with increased oxidative stress. Photobiomodulation (PBM) may impact wound healing processes by affecting the signaling pathways and molecules pertinent to tissue repair. In the present study, the effect of PBM (wavelength: 660 nm; energy density: 5 J/cm^2^) on levels of cox-2, IL-6, and TNF-*α* was determined in fibroblast cell culture models. Four WS1 models (normal, normal wounded, diabetic, and diabetic wounded) were irradiated at 660 nm, and the culture media was collected at 0, 24, and 48 h postirradiation. Cells that were not irradiated (0 J/cm^2^) served as the controls. The following parameters were determined postirradiation: cell morphology using light microscopy, cell viability using the Trypan Blue exclusion assay, and levels of the inflammatory markers cox-2, IL-6, and TNF-*α* were measured using ELISA. Cell migration increased in the wounded groups over the 48 h interval after PBM; viability improved postirradiation in the diabetic wounded groups at 0 and 24 h (*P* ≤ 0.05 and *P* ≤ 0.01, respectively); levels of cox-2 decreased in normal and diabetic wounded groups at 0 h (*P* ≤ 0.001) and increased in the diabetic and diabetic wounded groups at 48 h postirradiation (*P* ≤ 0.05 and *P* ≤ 0.01, respectively), while levels of IL-6 decreased in the normal (*P* ≤ 0.01), diabetic (*P* ≤ 0.05), and diabetic wounded (*P* ≤ 0.001) groups at 24 h and in the diabetic and diabetic wounded groups at 48 h (*P* ≤ 0.05) postirradiation. TNF-*α* was decreased in the normal wounded groups (*P* ≤ 0.05) at 48 h. Through its effect on decreased IL-6 levels in diabetic cell models, PBM at 660 nm may be successful at decreasing oxidative stress; however, the present study also found an increase in cox-2 levels at 48 h postirradiation.

## 1. Introduction

Photobiomodulation (PBM) uses light in the form of LASERs (light amplification by stimulated emission of radiation) and LEDs (light-emitting diodes) to stimulate and affect physiological processes in organisms at the molecular and cellular level. PBM has been shown to affect numerous pathways and molecules important to the wound healing process, and recently, our lab has shown that PBM controls the transcription of genes and stimulates cellular signaling in diabetic wounds [1, 2]. Wound healing comprises overlapping processes that occur in a certain sequence to ensure efficient tissue repair. The inflammatory response occurs during the early stages of wound healing (typically between days 1 and 3) and is initiated by molecular signals released by damaged tissue. The result is the initiation of biochemical signaling pathways ultimately aiming to heal the injured tissue [3, 4].

Injured cells release chemokines stimulating the chemotaxis of leukocytes into the area by binding to selective receptors. Cytokines released by the activated leukocytes cause inflammation [5]. Vascular responses in the inflammatory phase result in redness, edema, and warmth, while nerve stimulation leads to pain. This phase is important in keeping infectious agents at bay while promoting healing [6]. Leukocyte activity within the area eliminates microorganism threat and clears the area of debris [3, 7]. The macrophages that filtrate into the wounded area release a variety of cytokines including interleukin- (IL-) 1 beta, IL-6, IL-8, and tumour necrosis factor *α* (TNF-*α*) [4]. This in turn leads to cyclooxygenase (cox) 2 expression [8]. Cyclooxygenase, or prostaglandin synthase, consists of the enzymes cox-1 and cox-2 that are responsible for metabolizing or catalysing arachidonic acid to prostaglandins, prostacyclins, and thromboxanes [9]. These enzymes are regarded as proinflammatory and play a role in homeostatic and pathological systems. While prostacyclin and thromboxane A2 cause vasoconstriction necessary for hemostasis, prostaglandins (E and I series) contribute to inflammation by increasing vascular permeability and stimulating inflammatory cells [10, 11]. The overexpression of cox-2 stimulates the activation of metalloproteinases resulting in direct destruction of the extracellular matrix [12], and its overexpression thus contributes to wounds that do not heal timeously.

Inflammatory cytokines released by macrophages and neutrophils such as IL-6 and TNF-*α* stimulate inflammation by interacting with their respective receptors. Once bound to the receptor, intracellular signaling pathways are stimulated resulting in inflammation [4, 5]. In normal wound healing, apoptosis of cells involved in inflammation occurs. In order for wound healing to proceed optimally and efficiently, the inflammatory phase needs to conclude so that the other phases of wound healing may continue. In diabetes mellitus, the inflammatory phase continues indefinitely, and this, combined with various other factors, accounts for impaired wound healing [13]. The release of IL-6 and TNF-*α* is elevated in hyperglycemic states contributing to the sustained inflammatory response linked to delayed wound healing [14, 15]. Connective tissue of the dermis consists primarily of fibroblasts, and in tissue repair, their role in maintaining the structural integrity of the dermis is indispensable. Fibroblasts also release growth factors required for tissue regeneration, and in diabetic woundsdecreased fibroblast proliferation leads to wounds that do not heal well. In addition, when fibroblasts are stimulated, cytokines, chemokines, and prostanoids are produced [16].

Additionally, in diabetes, the interplay between inflammation and oxidative stress contributes to many of the problems associated with diabetes including foot ulcers that do not heal [17, 18]. Oxidative stress occurs due to the accumulation of oxygen-free radicals, and in diabetes, enhanced glucose metabolism accounts for the rise in oxidative stress. Free radicals are produced mainly by the mitochondria as metabolites or through the NADPH oxidases in cells and are required for normal physiological processes such as cell growth, differentiation, apoptosis, and activation of certain transcription factors [19]. The antioxidant system is necessary in eliminating excess free radicals. If the balance between the two is impaired in favour of the free radicals, pathological states arise [18, 20]. Increased levels of reactive oxygen species (ROS) contribute to increased levels of inflammatory cytokines, and enhanced inflammation can further the production of oxidative stress, thus, having an unfavourable effect on cells and tissues [21]. Esposito and colleagues suggest that in hyperglycaemia, an oxidative mechanism is a likely source for the production of IL-6 and TNF-*α* [22]. The authors found that the antioxidant glutathione impeded any further increase in IL-6 and TNF-*α*, thus, indicating that the levels of these cytokines increase with oxidative stress [22]. Activated cox-2 also leads to the production of ROS in mechanically wounded keratinocytes through the ROS-cox-2/PGE_2_ pathway [23].

PBM therapy and PBM has been shown to affect inflammation, inflammatory markers and cytokine production, and oxidative stress [24–27]. In the present study, we investigated the levels of cox-2, IL-6, and TNF-*α* postirradiation at 660 nm in diabetic wounded fibroblast cell culture models.

## 2. Materials and Methods

### 2.1. Cell Culture Models

Ethical clearance was obtained from the University of Johannesburg's Faculty of Health Sciences Research Ethics Committee (REC-01-98-2017). Four models of WS1 human skin fibroblast cells (ATCC®, CRL-1502™) were used in the study: normal (N), normal wounded (NW), diabetic (D), and diabetic wounded (DW). Cells were grown in Eagles Minimum Essential (MEM) medium containing 1.5 g/L sodium bicarbonate and Earls balanced salts. As previously described, D-glucose (17 mM/L) was added to MEM to achieve the diabetic models [28]. The media was changed twice weekly, and once cells reached a confluence of 80-90%, they were seeded in 3.4 cm diameter tissue culture plates at a density of 6 × 10^5^ cells per plate. Cell attachment to the tissue culture plates occurred when incubated at 37°C in 5% CO_2_. Prior to irradiation, culture media was discarded, and cells were rinsed with prewarmed Hanks Balanced Salt Solution and then replaced with 1 mL fresh media. To achieve the wounded model, a central scratch was made in the confluent cellular monolayer using a sterile 1 mL pipette [28]. Before the cells were irradiated, the tissue culture plates were incubated for 30 min and cell culture media collected at 0, 24, and 48 h postirradiation. Cell culture media was stored at -80°C and used within 6 weeks of collection.

### 2.2. Laser Irradiation

All lasers were supplied and set up by the National Laser Centre (NLC), Council for Scientific and Industrial Research (CSIR). Laser parameters are provided in Table 1. Previous studies in the same cell models have shown that a wavelength of 660 nm and a fluence of 5 J/cm^2^ have stimulated diabetic wounded cells [29–31]. Thus, in this study, a diode laser emitting at a wavelength of 660 nm and a fluence of 5 J/cm^2^ was used. Nonirradiated cells (0 J/cm^2^) served as controls. To prevent nuisance variables suggestive of polychromatic light that would normally interfere with the laser effect, laser irradiation took place in the dark and from above with the absence of the tissue culture dish lid.

### 2.3. Cell Morphology and Viability

Morphological changes in the cells were determined using light microscopy (Olympus CKX41). The Trypan Blue exclusion assay was used to determine cell viability. Equal volumes of Trypan Blue dye (0.4%; Sigma-Aldrich T8154) and cell suspension (10 *μ*l) were pipetted together and allowed to briefly stain. The mixture was loaded onto a Countess™ Cell Counting Chamber Slide and counted on the Invitrogen Countess™ II FL Automated Cell Counter immediately. Nonviable cells have compromised cell membranes and therefore stain blue as the dye enters the cells. Viable cells with undamaged cell membranes remain clear.

### 2.4. Enzyme-Linked Immunosorbent Assay (ELISA)

We used ELISA kits to determine levels of cox-2 (Sigma-Aldrich™ RAB1034), IL-6 (Sigma-Aldrich™; RAB0306), and TNF-*α* (Sigma-Aldrich™; RAB0476) according to the manufacturer's instructions. Briefly, 100 *μ*L of each standard and 100 *μ*L of sample (thawed culture media) were added into the wells of a 96-well plate. The plate was covered and left overnight at 4°C. The standard and samples were discarded and the plate washed four times with wash solution. The detection antibody (100 *μ*L) was then added to each well, and the plate was incubated for 1 h at room temperature with gentle shaking. Once the plate was washed four times with wash solution, 100 *μ*L of HRP-streptavidin was added to each well. The plate was incubated for 45 min at room temperature. The plate was again washed four times with the wash solution, and 100 *μ*L of the ELISA colorimetric TMB reagent was added to each well. The plate was incubated for 30 min at room temperature in the dark. Stop solution was added, and the plate was read at 450 nm immediately on the Victor3 multiplate reader (Perkin-Elmir).

### 2.5. Statistical Analysis

The experiments were performed three times (*n* = 3), and all assays were prepared in duplicate. The paired *t*-test was used to determine if any statistical differences existed between the nonirradiated (0 J/cm^2^) and the irradiated (5 J/cm^2^) groups within each model (normal, normal wounded, diabetic, and diabetic wounded) at the different time intervals. To determine if PBM at 660 nm had an effect on the overall levels of cox-2, IL-6, and TNF-*α* at 0, 24, and 48 h postirradiation, irrespective of model, a two-way ANOVA with a Tukey's post hoc test was used. Results are demonstrated as mean ± SD, and *P* ≤ 0.05 was considered significant. Statistics were performed using IBM® SPSS® Statistics for Windows Version 26 (IBM Corp., Armonk, NY, USA).

## 3. Results

### 3.1. Cell Morphology

**Figure 1:**
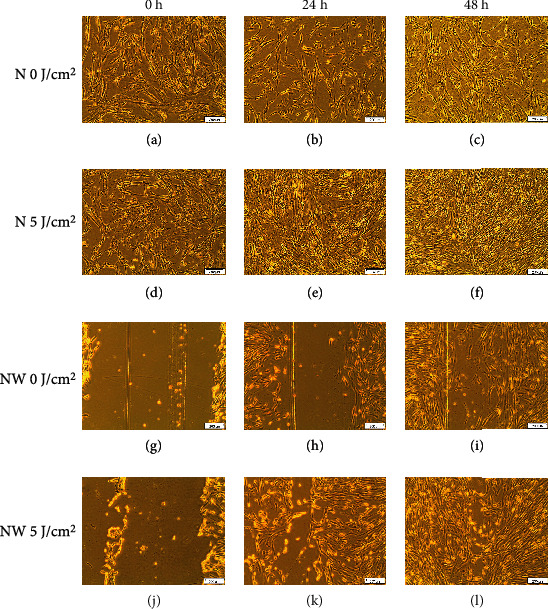
Morphology of normal (N) and normal wounded (NW) fibroblasts irradiated at 660 nm with 5 J/cm^2^ and measured over various time intervals. Nonirradiated cells (0 J/cm^2^) served as controls.

**Figure 2:**
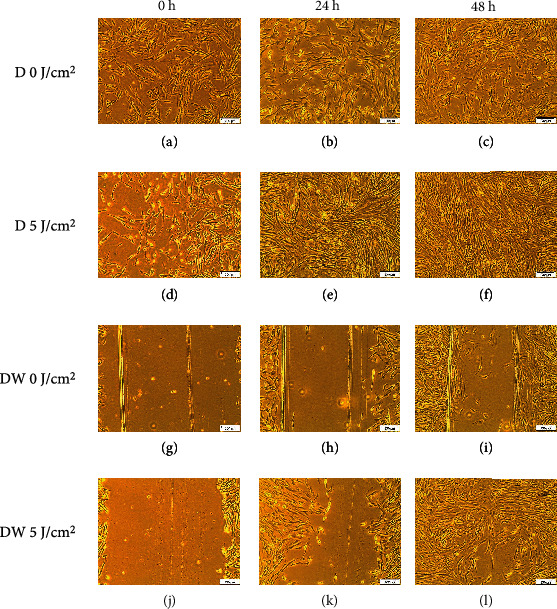
Morphology of diabetic (D) and diabetic wounded (DW) fibroblasts irradiated at 660 nm with 5 J/cm^2^ and measured over various time intervals. Nonirradiated cells (0 J/cm^2^) served as controls.

Morphological changes in WS1 cells and the wound gap width, created by the central scratch, are demonstrated in Figures 1 and 2. There were no noticeable morphological changes in the nonirradiated and irradiated cells over the 48 h period in both normal (Figure 1) and diabetic (Figure 2) groups. Fibroblast proliferation increased in cells exposed to irradiation (5 J/cm^2^). In the wounded groups, wound closure occurred faster in the irradiated groups (Figures 1 and 2(g)–2(l)). All cells grew as a monolayer and displayed strong adhesion properties.

### 3.2. Cell Viability

The Trypan Blue exclusion assay is used to determine the approximate percentage of viable (or live) and nonviable (or dead) cells in a sample.  Table 2 represents the percentage viability of cells. Cell viability significantly increased in the diabetic wounded irradiated group at 0 and 24 h when compared to the nonirradiated groups.

### 3.3. ELISA

**Figure 3:**
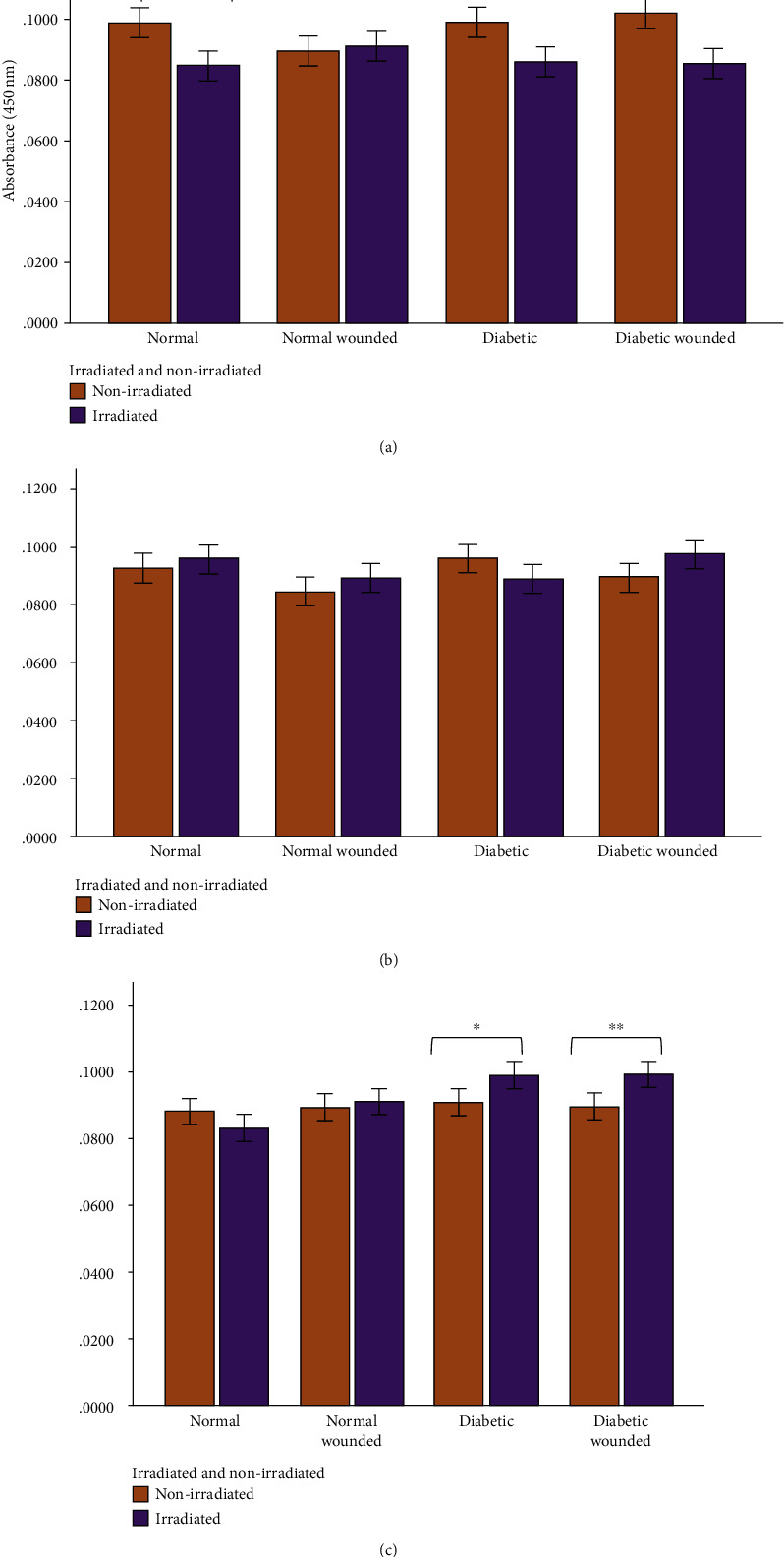
Human cox-2 levels in WS1 fibroblasts in normal, normal wounded, diabetic, and diabetic wounded cells irradiated at 660 nm with 5 J/cm^2^ and measured at (a) 0 h, (b) 24 h, and (c) 48 h postirradiation. Nonirradiated cells (0 J/cm^2^) served as controls. Results represent the mean of three repeats (*n* = 3) ± standard deviation. The paired *t*-test was used to determine differences in each model between the irradiated cells and the control. ^∗^*P* ≤ 0.05; ^∗∗^*P* ≤ 0.01; ^∗∗∗^*P* ≤ 0.001.

**Figure 4:**
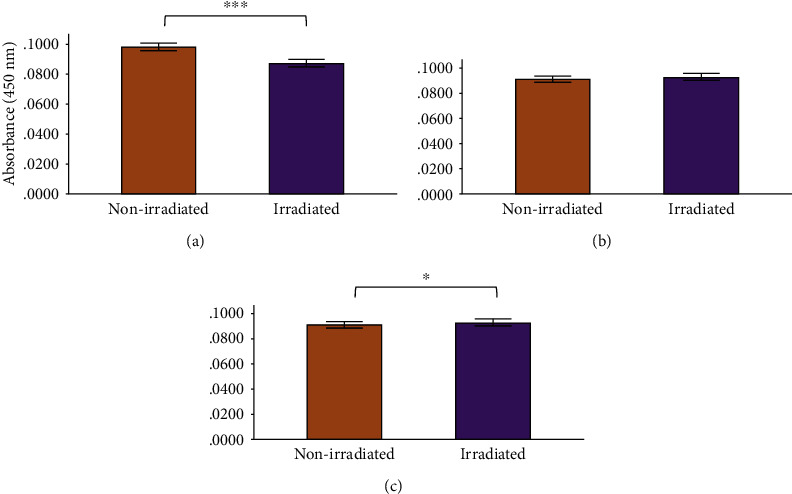
Human cox-2 levels in WS1 fibroblasts receiving no irradiation (0 J/cm^2^) or irradiation (5 J/cm^2^) at 660 nm at (a) 0 h, (b) 24 h, and (c) 48 h postirradiation. Results represented as the mean ± standard deviation. ^∗^*P* ≤ 0.05; ^∗∗^*P* ≤ 0.01; ^∗∗∗^*P* ≤ 0.001.

**Figure 5:**
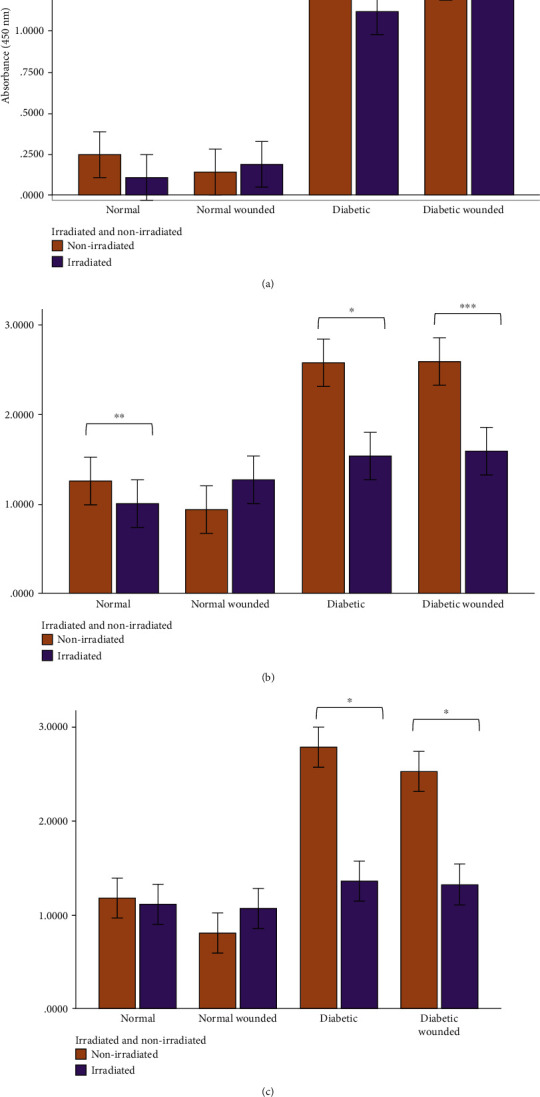
Human IL-6 levels in WS1 fibroblasts in normal, normal wounded, diabetic, and diabetic wounded cells irradiated at 660 nm with 5 J/cm^2^ and measured at (a) 0 h, (b) 24 h, and (c) 48 h postirradiation. Nonirradiated cells (0 J/cm^2^) served as controls. Results represent the mean of three repeats (*n* = 3) ± standard deviation. The paired *t*-test was used to determine differences in each model between the irradiated cells and the control. ^∗^*P* ≤ 0.05; ^∗∗^*P* ≤ 0.01; ^∗∗∗^*P* ≤ 0.001.

**Figure 6:**
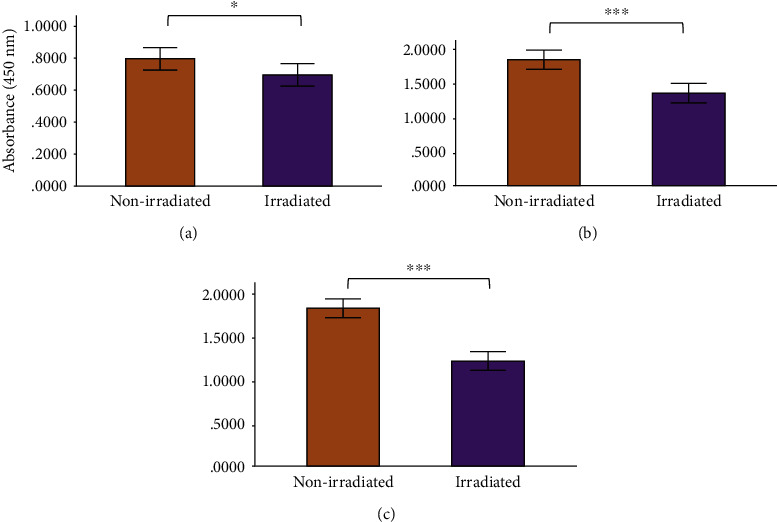
Human IL-6 levels in WS1 fibroblasts receiving no irradiation (0 J/cm^2^) or irradiation (5 J/cm^2^) at 660 nm at (a) 0 h, (b) 24 h, and (c) 48 h postirradiation. Results are represented as the mean ± standard deviation. ^∗^*P* ≤ 0.05; ^∗∗^*P* ≤ 0.01; ^∗∗∗^*P* ≤ 0.001.

**Figure 7:**
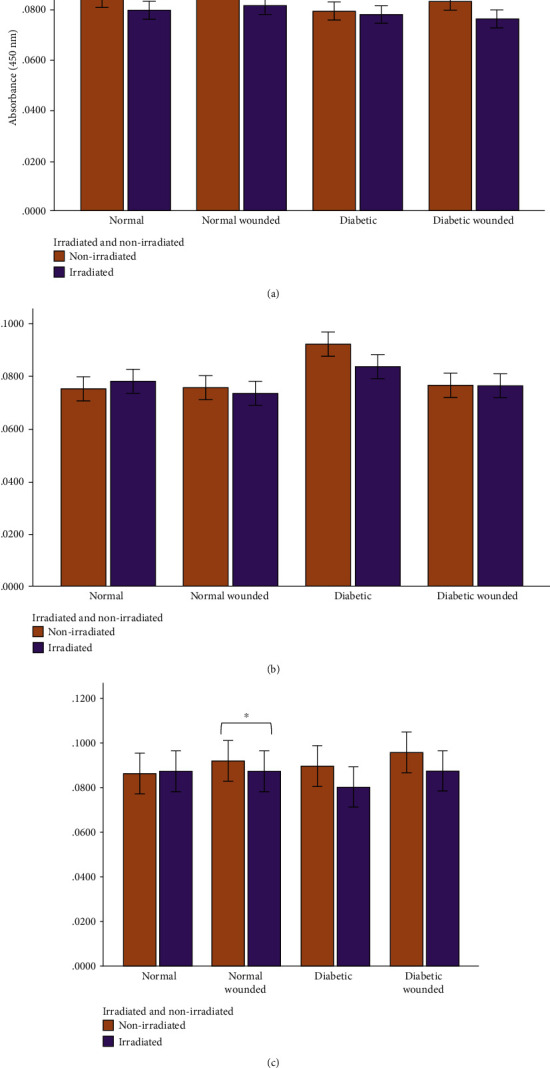
Human TNF-*α* levels in WS1 fibroblasts in normal, normal wounded, diabetic, and diabetic wounded cells irradiated at 660 nm with 5 J/cm^2^ and measured at (a) 0 h, (b) 24 h, and (c) 48 h postirradiation. Nonirradiated cells (0 J/cm^2^) served as controls. Results represent the mean of three repeats (*n* = 3) ± standard deviation. The paired *t*-test was used to determine differences in each model between the irradiated cells and the control. ^∗^*P* ≤ 0.05; ^∗∗^*P* ≤ 0.01; ^∗∗∗^*P* ≤ 0.001.

**Figure 8:**
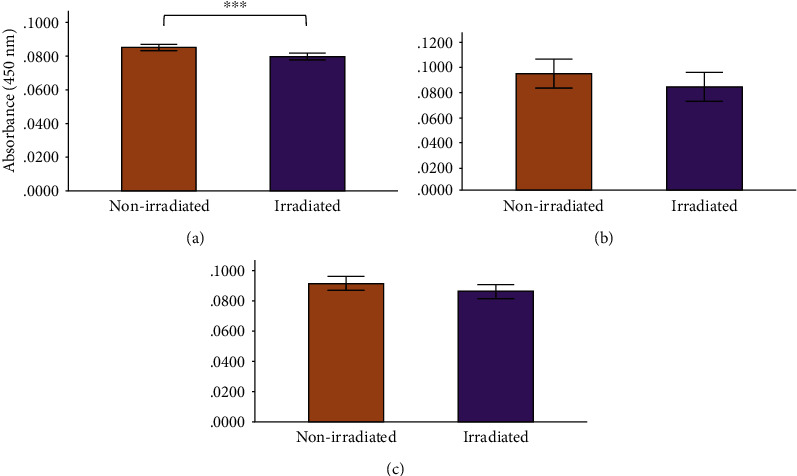
Human TNF-*α* levels in WS1 fibroblasts receiving no irradiation (0 J/cm^2^) or irradiation (5 J/cm^2^) at 660 nm at (a) 0 h, (b) 24 h, and (c) 48 h postirradiation. Results are represented as the mean ± standard deviation. ^∗^*P* ≤ 0.05; ^∗∗^*P* ≤ 0.01; ^∗∗∗^*P* ≤ 0.001.

Enzyme-linked immunosorbent assays were performed to measure the levels of cox-2, IL-6, and TNF-*α* in the nonirradiated and irradiated groups, and results are shown in Figures 3–8. Cox-2 levels initially decreased (at 0 h) between nonirradiated and irradiated cells in the normal (*P* = 0.007) and diabetic wounded (*P* = 0.009) groups; however, levels of cox-2 increased in both diabetic (*P* = 0.016) and diabetic wounded (*P* = 0.005) groups at 48 h postirradiation (Figure 3). Statistics for the two-way ANOVA showed that PBM significantly decreased cox-2 levels at 0 h (*P* ≤ 0.001); however, there was an increase in cox-2 levels at 48 h postirradiation (*P* = 0.013) (Figure 4).

IL-6 levels decreased in the diabetic group after irradiation (5 J/cm^2^; 660 nm) at 0 h, although the change was not significant. The most noticeable differences in IL-6 levels occurred 24 h postirradiation where a statistically significant decrease in IL-6 levels was measured in the normal (*P* = 0.006), diabetic (*P* = 0.016), and diabetic wounded (*P* = 0.001) groups (Figure 5). Furthermore, IL-6 levels decreased substantially in the diabetic group (*P* = 0.02) and in the diabetic wounded group (*P* = 0.05) 48 h postirradiation. At 0, 24, and 48 h, the levels of IL-6 were significantly higher in both the diabetic and diabetic wounded groups as compared to the normal and normal wounded groups, respectively (*P* ≤ 0.001), and this is indicative of an increased oxidative stress in the models receiving 17 mmol/L D-glucose. Statistics for the two-way ANOVA showed that PBM was successful at significantly decreasing the levels of IL-6 at 0 h (*P* = 0.041), 24 h (*P* ≤ 0.001), and 48 h (*P* ≤ 0.001) postirradiation (Figure 6).

The only significant decrease in TNF-*α* levels occurred in the irradiated normal wounded group at 48 h (*P* = 0.034). TNF-*α* also decreased in the experimental diabetic and diabetic wounded groups at 48 h postirradiation, although no statistical significance was reached (Figure 7). Statistics for the two-way ANOVA showed that PBM decreased the levels of TNF-*α* at 0 h (*P* ≤ 0.001); however, the decrease was not significant at 24 and 48 h postirradiation (Figure 8).

## 4. Discussion

In normal wounds, tissue repair follows a chronological sequence of events beginning with hemostasis and inflammation. The inflammatory phase serves as a protective essential step in order for tissue repair to successfully proceed. In wounds that display ineffective healing, such as those wounds in diabetics, the inflammatory stage of tissue repair becomes persistent and ineffective at protecting the wound from possible infections, and impaired wound healing in diabetes often leads to diabetic foot ulcers [13]. High glucose levels, through the intake of glucose, prompts oxidative stress through ROS and inflammation due to the expression of TNF-*α* in mononuclear cells, and it is suggested that these factors contribute to the proinflammatory state in diabetic wounds [13, 32–34]. In addition, high glucose stimulates the expression of IL-6 in mice [33]. In diabetic wounds, the increase in the cox-2/PGE_2_ pathway in macrophages leads to inflammation and decreased phagocytosis [35]. Additionally, an increase in ROS and elevated oxidative stress has also been associated with diabetes [36]. As prolonged inflammation and oxidative stress are linked to unsuccessful wound healing in patients with diabetes, we investigated the effects of PBM on the proinflammatory cytokines IL-6 and TNF-*α*, as well as the proinflammatory mediator cox-2 in diabetic wounded fibroblast cell culture models. Our results show that PBM at 660 nm and 5 J/cm^2^ decreased cox-2 levels immediately after irradiation in the normal and diabetic wounded groups; however, levels of cox-2 seemed to increase in diabetic and diabetic wounded groups 48 h postirradiation (Figures 3 and 4). The present results also show that PBM at 660 nm and 5 J/cm^2^ can significantly decrease the levels of IL-6 in diabetic and diabetic wounded models at 24 and 48 h postirradiation and decrease TNF-*α* levels in the diabetic and diabetic wounded models at 48 h postirradiation; however, this decrease was not statistically significant (Figures 5–8). The wound gap that was created by a central scratch also improved in the normal wounded and diabetic wounded groups postirradiation across a 48 h period (Figures 1 and 2).

In response to injurious stimuli, histamines, leukotrienes, and prostaglandins (PGs) are released due to mast cell activation and degranulation. Furthermore, when tissue injury occurs, leukotrienes and PGs are produced by the breakdown of membrane lipids by activated phospholipase A_2_ releasing arachidonic acid, the precursor for these eicosanoids [37]. Arachidonic acid is metabolized by two important enzymes: lipoxygenases producing the leukotrienes and cyclooxygenases- (cox-) 1 and -2 producing prostanoids (PGs, prostacyclins, and thromboxanes) [38]. PGs include prostaglandin E_2_, prostacyclin, prostaglandin D_2_, and prostaglandin F2*α* [39]. While cox-1 is important for normal physiological processes, both isoforms of cox lead to prostanoid production during inflammation; however, cox-2 is the more significant enzyme leading to prostanoid production in inflammation [39]. Activated cox-2 leads to vascular changes and leukocyte infiltration due to the effects of PGE_2_ in wound healing. Vasodilation and increased capillary permeability occur due to the action of PGE_2_ leading to the typical signs of inflammation: redness and swelling, while the effect of PGE_2_ on sensory neurons leads to the sensation of pain [39, 40].

In the current study, a decrease in human cox-2 levels in normal and diabetic wounded cells occurred at 0 h postirradiation. Interestingly, an increase in cox-2 was noted in the diabetic and diabetic wounded group at 48 h postirradiation. Petrellis and colleagues found that the gene expression of cox-2 increased with a 1 J energy dose among the various treatment and control groups in Walker tumour-induced Wistar rats [41]. The increase in cox-2 levels postirradiation is not easy to explain currently as the literature states that cox-2 is implicated in the inflammatory response; however, it is possible that cox-2 may be involved in decreasing inflammation through the production of anti-inflammatory prostaglandins but literature on this is scarce.

In response to injury, M1 phenotype macrophages release IL-6 and TNF-*α*, and in Type 2 diabetes mellitus, levels of these cytokines are elevated [3, 15]. TNF-*α* in particular plays a vital role in insulin resistance and tissue inflammation [42, 43]. Both forms of TNF-*α*, the soluble form and its precursor transmembrane form, play a pivotal role in inflammation once bound to TNF-*α* receptors [44]. IL-6 is released by a variety of cells including fibroblasts, and it has many functions in addition to promoting inflammation [45].

In the present study, TNF-*α* levels decreased in the irradiated (experimental) diabetic models at 24 and 48 h, and in the irradiated (experimental) diabetic wounded models at 0 and 48 h, however, the differences did not reach statistical significance. Our study also found that levels of the IL-6 cytokine decreased significantly in irradiated diabetic and diabetic wounded groups at both 24 h and 48 h. This finding is similar to Petrellis et al. who reported that at the same wavelength of 660 nm, IL-6 and TNF-*α* levels were significantly reduced in their tumour experimental groups, whereas an increase in the levels of these cytokines were recorded in the groups receiving an energy dose of 3 J and 6 J [41]. Salehpour et al. found a reduction in serum IL-6 and TNF-*α* levels in the neural tissues of mice after receiving 5 days of PBM at 810 nm and 33.3 J/cm^2^. These differences were noted in the groups that received PBM only, coenzyme Q10 only, and when a combination of both was used [26]. Our results are contradictory to Fernandes et al. who found an increase in IL-6 production (and IL-6 mRNA expression) in J774 macrophage-like cells irradiated with a 660 nm laser. The increase in IL-6 levels could not easily be explained by the researchers; however, the study found a decrease in TNF-*α* expression, and a downregulation of TNF- *α* and cox-2 protein production in the J774 cells irradiated at 660 nm (7.5 J/cm^2^) and 780 nm (2.6 J/cm^2^), with the latter wavelength causing more of a decrease [25].

An elevation in ROS generation after PBM at 660 nm *in vitro* and *in vivo* was noted by Rupel and colleagues, whereas ROS generation decreased when other wavelengths of light were used in their study [46]. Our lab recently showed that in diabetic fibroblast models, PBM at 660 nm and 5 J/cm^2^ is effective in decreasing oxidative stress through the inhibition of the FOXO1 signaling pathway [31]. The current study provides a possible alternative mechanism through which PBM may be effective at decreasing oxidative stress by lowering the levels of IL-6 in diabetic models; however, a study evaluating the effect of PBM at 660 nm on both IL-6 and oxidative stress is required to confirm this.

## 5. Conclusions

In conclusion, the present study shows that PBM at 660 nm and 5 J/cm^2^ decreases the levels of the proinflammatory cytokine IL-6 in diabetic wounded WS1 fibroblasts, and this decrease is most apparent at 48 h postirradiation. The data is consistent with most of the studies on the effects of PBM using various wavelengths on IL-6. Interestingly, our study found an initial decrease in cox-2 levels, which then increased at 48 h postirradiation, but more studies are needed to determine if and why this increase may occur. In the present study, levels of TNF-*α* decreased at 48 h in the diabetic and diabetic wounded groups; however, the differences did not reach statistical significance.

## Figures and Tables

**Table 1:** Laser parameters using the 660 nm diode laser.

Laser parameter	
Laser type	Diode
Wavelength (nm)	660
Wave emission	Continuous
Power output (mW)	101.25 ± 5.91
Power density (mW/cm^2^)	11.15 ± 0.65
Fluency (J/cm^2^)	5
Energy (J)	45.53 ± 0.15
Spot size (cm^2^)	9.1
Irradiation time (± min, s)	7 min and 29 s ± 24.56 s

**Table 2:** The Trypan Blue exclusion assay was performed to assess cellular viability (%) of nonirradiated (0 J/cm^2^) and irradiated cells (5 J/cm^2^) at 0, 24, and 48 h postirradiation. An increase in cell viability was noted in the diabetic wounded group at 0 and 24 h postirradiation.

	Nonirradiated cells (0 J/cm^2^) % viability (± SD)	Irradiated cells (5 J/cm^2^) % viability (± SD)
Normal		
0 h	89 (±3)	89.67 (±5.5)
24 h	66 (±7)	77.33 (±6.506)
48 h	91 (±2.646)	86.33 (±1.528)
Normal wounded		
0 h	92 (±6.08)	92.67 (±2.08)
24 h	71.67(±10.066)	76 (±4.359)
48 h	74.67 (±7.234)	86 (±2)
Diabetic		
0 h	55.33 (±4.041)	59 (±3.464)
24 h	70 (±6)	78.33 (±4.619)
48 h	82 (±6.254)	89.67 (±2.517)
Diabetic wounded		
0 h	57 (±3.464)	73 (±8.718)^∗^
24 h	58.33 (±3.055)	83 (±5.292)^∗∗^
48 h	90.33 (±2.309)	90.33 (±3.215)

^∗^ *P* ≤ 0.05; ^∗∗^*P* ≤ 0.01.

## Data Availability

Data is available on request.
